# How Positivity Links With Job Satisfaction: Preliminary Findings on the Mediating Role of Work-Life Balance

**DOI:** 10.5964/ejop.v11i3.869

**Published:** 2015-08-20

**Authors:** Hod Orkibi, Yaron Ilan Brandt

**Affiliations:** a Graduate School of Creative Arts Therapies, University of Haifa, Haifa, Israel; Aalborg University, Aalborg, Denmark; NEOMA Business School, Reims, France

**Keywords:** work-life balance, positive psychology, job satisfaction, positivity

## Abstract

The positive characteristics that can help people juggle their work and personal roles and experience greater job satisfaction are attracting increased research attention. This study presents a conceptual model to account for the association between employees’ positive orientation (i.e., the tendency to evaluate self, life, and the future in a positive way) and their job satisfaction (N = 108). As theorized, the results indicate that employees’ ability to manage their work-life balance fully mediates the relation between their positive orientation and job satisfaction. This suggests that a positive orientation serves as an adaptive personal resource that can facilitate employees’ ability to balance work and non-work demands and hence can foster job satisfaction. The practical implications for positive psychological interventions in organizational settings are discussed.

Most employees face the challenge of balancing work and personal demands (Baltes, Clark, & Chakrabarti, 2010) to sustain efficiency in both domains and to protect their well-being (Linley, Harrington, & Garcea, 2010; Lunau, Bambra, Eikemo, van der Wel, & Dragano, 2014). It comes as no surprise that increasing attention has been paid to individual factors associated with positive outcomes in work and personal life, including people's self-esteem, life satisfaction, and optimism. Although these three factors are highly and positively correlated, most studies have focused on the association of each with specific outcomes.

For example, high self-esteem, which refers to how people regard and accept themselves (Harter, 1993), has been associated with more effective coping approaches and persistence when facing problems and difficulties (Kernis, 2003), and with having a greater sense of control over life events (Tedeschi & Norman, 1985). Life satisfaction, which refers to an overall evaluation of one’s life as satisfying (Diener, Emmons, Larsen, & Griffin, 1985), has been associated with positive coping strategies (Jones, Rapport, Hanks, Lichtenberg, & Telmet, 2003), internal locus of control (Yeung & Chow, 2000), and better mental and physical health (Siahpush, Spittal, & Singh, 2008). Likewise, optimism, which refers to individuals’ general expectations to experience more good than bad things in their future, has been associated with desirable outcomes and health through active and effective coping in the face of hardships (Rasmussen, Wrosch, Scheier, & Carver, 2006; Scheier & Carver, 2003).

Nevertheless, an accumulating body of research suggests that self-esteem, life satisfaction, and optimism are at the core of a single trait-like construct termed *positive orientation* (hereafter: *positivity*); i.e., a basic predisposition underlying individuals’ evaluations of self, life, and the future in a positive way (Heikamp, Alessandri, Laguna, Petrovic, Caprara, & Trommsdorff, 2014). The structure of this construct has been supported in studies showing cross-cultural stability in Western and Asian (i.e., Japan) samples (Caprara, Alessandri, Trommsdorff, Heikamp, Yamaguchi, & Suzuki, 2012), genetic twin studies (Caprara et al., 2009), and longitudinal studies (Alessandri, Caprara, & Tisak, 2012a). Furthermore, positivity has predicted outcomes related to personal and interpersonal adjustment, including high positive affect and low negative affect, better self-reported health and perceived quality of friendship (Alessandri, Caprara, & Tisak, 2012b). In a work-related study, positive orientation significantly predicted in-role and extra-role performance (organizational citizenship behaviors) (Alessandri, Vecchione, Tisak, Deiana, Caria, & Caprara, 2012).

Although the benefits associated with positivity seem clear, the ways in which positivity translates into job-related outcomes is largely understudied and unknown. Scholars have argued that *how* positivity relates to job-related outcomes “represents a critical question since it may lead to more effective interventions and to successful strategies for employees to fully develop their potentials” (Alessandri, Borgogni, Schaufeli, Caprara, & Consiglio, 2015, p. 2). To address this gap in the literature, this study presents a conceptual model that captures the mechanisms through which positivity translates into job satisfaction, which is defined as how individuals feel about their jobs and different aspects of their jobs, and the extent to which they like (*satisfaction*) or dislike (*dissatisfaction*) their work (Spector, 1997, p. 2). This relationship is particularly important given the strong impact of job satisfaction on indicators of physical and mental well-being (for a meta-analysis see Faragher, Cass, & Cooper, 2005).

This study also explores how personal resources exert their influence on other resources, which in turn can lead to more positive and adaptive outcomes (Alessandri et al., 2015; Hobfoll, 2011; Scheier & Carver, 2003). Consistent with this line of thought, we theorized that positivity (as a personal resource) should be associated with greater job satisfaction through its influence on individuals’ ability to manage work-life balance, defined as the right combination of participation in paid work and other aspects of one’s life (Hayman, 2005). In the following section, we present the theoretical lenses that contributed to hypothesis development.

## Positivity as an Adaptive Personal Resource

The positive orientation framework addresses positivity directly as a unidimensional construct, rather than indirectly through other related but distinct constructs such as self-esteem, life satisfaction, and optimism (Caprara, Alessandri, Trommsdorff, et al., 2012). According to this framework, “viewing oneself, life, and the future under a positive outlook attests to a basic predisposition that exerts an important biological function in making people prone to cope with life, despite adversities, failures, and loss” (Alessandri et al., 2015, p. 4). This reasoning is based on earlier cognitive theories that negative views of the self, the world, and the future characterize depressed individuals (Beck, 1967), whereas Caprara and colleagues conceptualized a positive orientation as an adaptive propensity underlying individuals’ positive evaluations towards self, life, and future (Caprara et al., 2009; Caprara, Steca, Alessandri, Abela, & McWhinnie, 2010).

The model presented here draws on this notion of positivity and two supporting theories. The first is the *conservation of resources theory*, which argues that personal resources (e.g., positivity) tend to generate gains in other resources, which in turn lead to greater well-being (Hobfoll, 1989, 2011). This leads to the supposition that more positive individuals are better equipped to balance work and non-work demands, such that positivity (as adaptive personal resource) should lead to greater work-life balance, and hence to greater job satisfaction. The model also draws on *self-regulation of behavior theory* according to which individuals who are self-confident and optimistic tend to expect positive outcomes across various life domains (Carver & Scheier, 1998; Scheier & Carver, 2003). Because they are confident about their success in the future, these people tend to exert a continuing effort and to succeed more than individuals who are less confident and optimistic. In addition, these individuals tend to use more highly active problem-focused coping strategies, and when these are not possible, they turn to adaptive emotion-focused strategies such as acceptance, humor, and positive reframing (Rasmussen, Wrosch, Scheier, & Carver, 2006). In contrast, less optimistic individuals tend to experience more doubt, adopt avoidant behavior, and consequently may give up and experience a sense of failure when trying to reach their goals.

Based on these two complementary theories, we theorized that individuals who are more positive should be more satisfied with their job, at least partly because they are better able to manage their work-life balance. In other words, we expected that work-life balance would mediate the relationship between positivity and job satisfaction.

## Work-Life Balance as a Mediator

Empirical research has amply documented the direct links between positivity and job-related outcomes but the specific direct and indirect associations between positivity and job-satisfaction are largely neglected. The concept of *work-life balance* focuses on minimizing the tensions between work and other parts of one’s life (Hayman, 2005; State Services Commission - New Zealand, 2005). This concept contrasts with *work-family balance*, which refers to the assessment of whether work and family resources are adequate to meet work and family demands, such that participation is effective in both domains (Voydanoff, 2005). *Work-family conflict* refers to incompatibilities between work and family responsibilities due to limited resources, such as time and energy (Greenhaus & Beutell, 1985). The current study employs the term work-life balance because it is also applicable to single individuals without a family, whereas the other two terms are, by definition, restricted to individuals with families.

Several studies have suggested that high self-esteem can help employees avoid stress and hardships as well as cope with challenges at work (Grandey & Cropanzano, 1999; Nikandrou, Panayotopoulou, & Apospori, 2008; Ruderman, Ohlott, Panzer, & King, 2002). Other studies have found significant associations between life satisfaction and work-life balance (Allen, Herst, Bruck, & Sutton, 2000; Fisher, 2002) and indicated that optimism helps employees achieve a better work-life balance (Allen, Johnson, Saboe, Cho, Dumani, & Evans, 2012; Aryee, Srinivas, & Tan, 2005; Luthans, Avolio, Avey, & Norman, 2007). With respect to the direct link between work-life balance and job satisfaction, studies have shown that when personal life interferes with work it is negatively linked to job satisfaction (Thomas & Ganster, 1995) and positively linked to distress at work (Frone, Russell, & Cooper, 1992). Similarly, when work interferes with personal life it is negatively linked to job satisfaction and other outcomes (Amstad, Meier, Fasel, Elfering, & Semmer, 2011; Cortese, Colombo, & Ghislieri, 2010).

Only a few studies have examined the role of work-life balance as a mediator, for example, as regards the relationship between job satisfaction and life satisfaction (Virick, Lilly, & Casper, 2007) or between work outcomes such as job satisfaction, organizational commitment, and career accomplishment (Marcinkus, Whelan-Berry, & Gordon, 2007). However, to the best of our knowledge, work-life balance has not been tested as a mediator in the specific relationship between positivity and job satisfaction. In this sense, the present study extends the literature by theorizing and examining whether a better work-life balance can be achieved by tapping personal resources such as positivity, and hence can translate into greater job satisfaction.

## Study Hypotheses

This study contributes to the literature in two major directions that are largely under-studied. It simultaneously explores the empirical relations among positivity, work-life balance, and life satisfaction, and examines whether the indirect relationship between positivity and job satisfaction is mediated through work-life balance. By conceptualizing positivity as an adaptive personal resource that has a pervasive influence on other resources, we hypothesized that (1) there would be a positive correlation between positivity, work-life balance, and job satisfaction; and that (2) the relationship between positivity and job satisfaction would be mediated by work-life balance.

## Method

### Participants and Procedure

One hundred and eight participants were recruited from several advertising and accounting organizations via e-mail. To encourage participation, contacts were told that three prizes would be raffled among participants in each organization. Those wishing to take part in the raffle were required to submit an e-mail address. All participants were asked to fill out an anonymous online questionnaire that measured work-life balance, job satisfaction, and positivity. Logging into the online questionnaire confirmed informed consent to participate in the study, but participants could withdraw at any time. The University Human Research Ethics Committee approved the study (approval no.081/13).

The sample was composed of employees in an accounting firm (*n* = 23; 21%) and employees in three different advertising firms (*n* = 85; 79%). A preliminary multivariate analysis of variance (conducted with workplace as the independent variable and job satisfaction, work-life balance, and positivity as the dependent variables) revealed no significant differences by workplace. Therefore, participants were treated as one sample in the remaining analyses. Of the entire sample, 60% were female and 40% were male. The mean age was 31 with an age range of 22 to 48 (most respondents were between 28 and 32). The majority of the respondents (93%) were born in Israel, 5% were born in the former Soviet Union, and the remaining 2% were born in Europe and other countries. The majority of the participants (98%) identified themselves as Jewish, and the remaining 2% identified themselves as Christians and members of other religions. Of the entire sample, 75% identified themselves as secular, 13% as traditional, 4% as religious-Zionist, and 8% as other. The majority of all respondents were married (51%), lived in the center of Israel (90%), rented an apartment with their roommates or partners (60%), and were currently childless (70%; number of children ranged from zero to three). The mean for years in the current job was 5, with a range of 2 months to 30 years. The majority of respondents held a Bachelor’s degree (60%), followed by non-degree diplomas (18%), Master’s degrees (15%), and others (7%).

### Measures

Respondents completed three self-report questionnaires in Hebrew that were translated into Hebrew from English by bilingual professionals using Brislin's back translation technique (Cha, Kim, & Erlen, 2007).

#### Work-Life Balance

**Figure 1:**
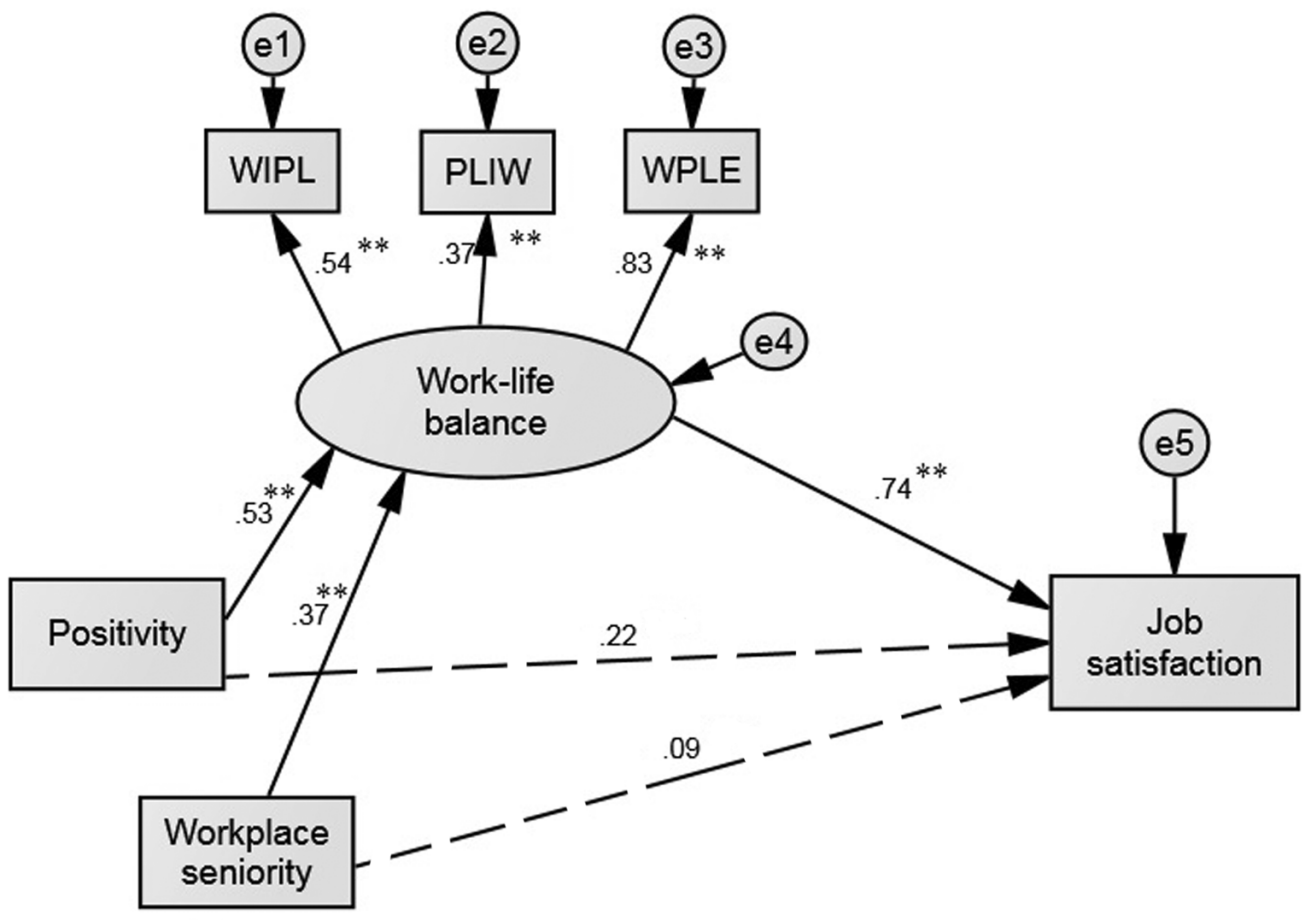
Model depicting the hypothesized mediating role of work-life balance in the relationship between Positivity and Job satisfaction. *Note.* Workplace seniority was inserted as a control variable. All coefficients are standardized and significant at the *p* < .01 level. Dashed paths are not significant.

A 15-item self-report scale (Hayman, 2005) was used to measure perceived work-life balance across three dimensions: work interference in personal life (WIPL, e.g., “I neglect personal needs because of work”), personal life interference with work (PLIW, e.g., “My work suffers because of my personal life”), and work-personal life enhancement (WPLE, e.g.,“I have a better mood at work because of my personal life”). Respondents indicated the frequency with which they had felt each item over the previous three months on a scale ranging from 1 (*not at all*) to 7 (*all the time*), where a higher score indicated greater work-life balance (WIPL and PLIW items were reverse coded to reflect balance because these dimensions are negatively worded and reflect “imbalance”). In the present study, the Cronbach’s alpha for WIPL was α = .89, PLIW was α = .78, WPLW was α = .68. The Cronbach’s alpha for the entire scale was α = .86, and the scale dimensions were used as indicators of a latent variable in the model (see Figure 1).

#### Job Satisfaction

A short version of Brayfield and Rothe’s (1951) scale was used to measure employees’ job satisfaction based on their attitudes towards their work (Judge, Locke, Durham, & Kluger, 1998). The scale had five items (e.g., “I feel fairly well satisfied with my present job”), scored on a scale ranging from 1 (*strongly disagree*) to 5 (*strongly agree*). Good internal consistency reliability was reported (α =.88). The correlation with the original Job Description Index (Smith, Kendall, & Hulin, 1969) was high (.89). The correlations between self-reports and significant-others reports show an average of .69 (Judge et al., 1998). In the present study, the Cronbach’s alpha for internal consistency was α = .73.

#### Positivity

The Positivity Scale is an 8-item instrument that assesses the general tendency for a positive orientation as a unidimensional construct. The scale’s psychometric properties have been well established in different samples and cultures, including convergent and discriminant validity, internal validity, and temporal stability (for details see Caprara, Alessandri, Eisenberg, et al., 2012). Recent results provide evidence for gender and cross-cultural invariance and construct validity of the scale in five different samples across Europe (Heikamp et al., 2014). Participants responded on a scale ranging from 1 (*strongly disagree*) to 5 (*strongly agree*). A sample item is: “I have great faith in the future.” Previous studies showed that the alpha coefficient for the scale's internal consistency ranged from .75 to .79, the mean corrected item-total correlation was .48 (*SD* =.06), and the determinacy coefficient was as high as .89. In the present study, the Cronbach’s α was .74.

### Data Analysis

Correlations were calculated to explore the relationships between all the variables. Using Amos software, structural equation analysis (SEM) was run to provide estimates of the mediation model. The mediating effect was tested using bootstrap analysis with a confidence level of 0.95 and bootstrap bias-corrected samples set at 5000 (Preacher & Hayes, 2004).

## Results

As shown in Table 1, the results confirmed the first hypothesis, indicating a positive correlation between positivity, work-life balance (total score), and job satisfaction.

**Table 1:** Means, Standard Deviations, and Correlations Between Study Variables (N = 108)

	*Mean*	*SD*	Positivity	WLB	JS
Positivity	3.84	0.45	–		
WLB	3.34	0.81	.35**	–	
JS	3.53	0.69	.19*	.46**	–

*Note.* WLB = Work-life balance; JS = Job satisfaction.

*p < .05. **p < .01.

An exploratory multivariate analysis of variance (MANOVA) was conducted with the demographic variables of interest as independent variables (age, gender, job title, number of children, marital status, and workplace seniority) with job satisfaction, work-life balance, and positivity as the dependent variables. The MANOVA revealed significant differences between employees with different seniority (years) in the workplace (Wilks’ Lambda = .770, *F*(12, 1270) = 2.58, *p* = .007). Significant positive correlations were found between workplace seniority and work-life balance (*B* = 0.048, *t* = 3.37, *p* < .001, η_p_^2^ =.098) and between seniority and job satisfaction (*B* = 0.027, *t* = 2.02, *p* < .005, η_p_^2^ =.037). Therefore, workplace seniority was included as a control variable in the mediation model.

### Mediation Analysis

SEM was used to test the mediating role of work-life balance (as a latent variable) on the relationship between positivity and job satisfaction. The bootstrap test method for indirect effects was used with a confidence interval (CI) level set at .95 and bootstrap bias-corrected samples set at 5000 (Preacher & Hayes, 2004). Acceptable fit parameters have been defined as *X* ^2^/*df* ≤ 3, a comparative fit index (CFI) ≥ .95, a Tucker–Lewis index (TLI) ≥ .95, and the root mean square error of approximation (RMSEA) < .080 (Schreiber, Nora, Stage, Barlow, & King, 2006).

The measurement model for work-life balance, assuming second-order structure with three factors, showed a good fit to the data: CMIN/DF = 1.75 (*p* < .00), CFI = .93, TLI = .91, RMSEA = .08. Further analysis of the mediation model in Figure 1 indicated a good fit with the observed data on the following fit indices: CMIN/DF = 1.51 (*p* = .16), CFI = .97, TLI = .93, RMSEA = .07. Bootstrap results provided support for the mediating role of work-life balance in the relationship between positivity and job satisfaction (*p* < .001, 95% CI [.187, 0.781]). Because the confidence interval did not include zero, the null hypothesis of no mediation could be rejected. The direct effect of positivity on job satisfaction was not significant (β = .22, *p* = .10), indicating full mediation. In addition, the total effect was not significant (*p* < .123, 95% CI [–.070, 0.589]). As seen in Figure 1, the path coefficients from positivity to work-life balance (β = .53, *p* < .001, 95% CI [.709, 0.318]) and from work-life balance to job satisfaction (β = .74, *p* < .001, 95% CI [1.152, 0.435]) were statistically significant; as positivity increased, work-life balance increased and as work-life balance increased, job satisfaction increased.

Because SEM only provides estimates of the total indirect effect between variables, we also used the recommended procedure to calculate each of the specific indirect effects within the model, which yielded a separate *z*-score for each indirect effect (Taylor, MacKinnon, & Tein, 2008). A specific indirect effect represents the portion of the total indirect effect that operates through a specific intervening variable within a model. The test confirmed that work-life balance mediated the relationship between positivity and job satisfaction (*z* = 2.70, *p* < .01).

## Discussion

Today, the role of positive psychological factors in helping individuals sustain optimal functioning in both work and personal life domains (Baltes et al., 2010) is attracting growing attention. This study contributes to the literature not only by shedding light on the relationships between personal positivity, work-life balance, and job satisfaction, but also by providing initial evidence for the psychological mechanism through which a trait-like positive psychological resource such as positivity might lead to greater job satisfaction. This strengthens the emerging body of literature indicating the critical pervasive role of positivity as an adaptive personal resource that exerts its influence on other resources.

Specifically, the findings lend empirical weight to previous studies showing that individuals’ tendency to evaluate self, life, and the future in a positive way has adaptive value across many domains of functioning, including work (e.g., Alessandri et al., 2015) and personal life (e.g., Alessandri et al., 2012b). Thus, individuals who have this type of positive orientation may be more likely to focus on the positive side of experiences; i.e., how work and personal life enhance each other rather than interfere with each other. This argument coincides with the idea that shifting the focus of attention by reinterpreting threats as challenges can be an adaptive way to conserve positive resources (Hobfoll, 1989, p. 519).

Hobfoll’s (1989, 2011) conservation of resources theory postulates that individuals are motivated to gain, obtain, retain, foster, and protect resources, as well as prevent resource loss. Resources not only tend to aggregate and generate gains of other resources, but individuals with greater resources are less vulnerable to resource loss and more capable of orchestrating resource gain. Hobfoll (2011) noted that gain cycles are critical to work-family interactions, as “both [are] jealous demanders of individuals’ resources, and to the extent that resources are built in one domain that facilitates the other domain, this ‘battle for resources’ can become a common agenda” (p. 118). In line with this view, the findings of this study imply that individuals with a positive orientation might be more likely to “orchestrate resource gain” in terms of being able to manage and cope with the challenges associated with “work interference with personal life” and “personal life interference with work” (Hayman, 2005). Thus, individuals with a positive orientation may experience a sense of mastery and achievement in balancing work and non-work demands. This positive experience may spill over and make them feel more satisfied with their jobs. The propensity for a positive orientation, therefore, may translate into greater job satisfaction because it facilitates the ability to balance work and non-work demands.

Furthermore, from a behavioral perspective, individuals with a positive orientation tend to succeed because they are more diligent in dealing with problems (Scheier & Carver, 2003). They make more effort, engage in more active problem-focused coping strategies, and therefore are more likely to reach their goals, or turn to adaptive emotion-focused strategies such as acceptance, humor, and positive reframing (Rasmussen, Wrosch, Scheier, & Carver, 2006). Thus, a positive orientation may act as a motivational force that sustains an individual’s efforts to balance work and non-work demands. A positive orientation may help people make a continuing effort to deal with work-life balance problems, use adaptive coping strategies that mitigate their perception of these problems, and consequently experience more job satisfaction.

Because individuals with a positive orientation see events as predictable and generally in their best interests, they may perceive events in their life and work as less threatening, their life and work related goals as more attainable, and be more resilient to the impact of the challenges and stressors resulting from daily experiences and social interactions. A positive orientation may thus also contribute to work-life balance by fostering high tolerance to stress, resiliency, and commitment to valued goals such as family quality time (cf. Grassi et al., 2014).

In short, our findings help to explain how having positive orientation toward one’s self, life, and future may act as an adaptive personal resource that exerts its influence on other resources, such as translating into greater job satisfaction by means of facilitating the ability to balance work and non-work demands.

### Limitations and Future Directions

There are several limitations to this study that deserve attention. First, the cross-sectional design of the study precludes inferences of causality. Future studies should include longitudinal data or interventions to examine work-life balance as a mechanism of treatment change. Second, because the data were collected from self-report measures, future studies should collect data from respondents’ co-workers, supervisors, and/or employers to enhance validity. A related possible limitation is that the data collected from respondents regarding their work-life balance were self-perceived in that experiencing work-life balance was subjective. Future studies should include a sample of employees’ actual work hours as well as reports of co-workers, supervisors, or family members. Although a pre-study sample size estimation was conducted, another limitation is the relatively small sample size and the focus on work in a specific industry. Future studies should include a larger sample and employees from different job sectors. Relatedly, the sample included respondents from the urban center of the country, which is typically characterized by a fast-paced lifestyle, compared to peripheral or rural areas. Future studies should include a more heterogeneous sample with greater variance in socio-demographic characteristics. Multi group SEM analysis could be used to compare the model invariance across different groups of employees (Byrne, 2004). In addition, because all the respondents were Israelis, and the vast majority were Jewish, future studies should include workers from other countries and religious affiliations to scrutinize the external validity of findings. Finally, the current study focused on the contribution of positivity to the relationship between job satisfaction and work-life balance. Future studies should examine other possible positive psychological variables that might contribute to this relationship as well, such as work social support, creativity, innovation, self-control, coping ability, and integrity.

### Implications

The model theorized in this study has practical implications for promoting employees’ potentials and strengths in balancing the demands of work and life, which may in turn lead to experiencing more satisfaction on the job. Based on the current results, organizations would be wise to assess employees’ positive orientation, their ability to manage work-life balance demands, and job satisfaction. Employees with a less positive orientation, difficulties balancing work-life demands, and poor job satisfaction could benefit from organizational interventions aiming at the amplification of a positive orientation and the skills needed to preserve the work-life balance.

Specifically, such interventions could employ psychological strategies for enhancing positive cognitions and emotions regarding one’s self, life, and future, which are the “ingredients” of positivity. A recent systematic review of research suggests that positive psychological interventions applied in the organizational context are a promising way to enhance employee well-being and performance, as well as to reduce stress, burnout, depression, and anxiety (see Meyers, van Woerkom, & Bakker, 2013). Nevertheless, future experimental studies should examine the extent to which implementing interventions that specifically aim at identifying, developing and broadening employees’ positive orientation can promote their ability to balance work-life demands and increase their job satisfaction.

### Conclusion

This study suggests that work-life balance may serve as a psychological mechanism that enables individuals to harness their positive orientation such that it translates into greater satisfaction with their job. Individuals with a positive orientation appear to have more resources to balance work and life demands that may lead to experiencing more job satisfaction. The model presented here should be tested in future experimental studies, because a poor work-life balance and low job satisfaction can influence not only employees’ performance but also their well-being.
